# Toxicokinetics of 2-ethylhexyl salicylate (EHS) and its seven metabolites in humans after controlled single dermal exposure to EHS

**DOI:** 10.1007/s00204-024-03827-x

**Published:** 2024-08-12

**Authors:** Laura Kuhlmann, Thomas Göen, Julia Hiller

**Affiliations:** Institute and Outpatient Clinic of Occupational, Social, and Environmental Medicine, Friedrich-Alexander-Universität Erlangen-Nürnberg, Henkestr. 9–11, 91054 Erlangen, Germany

**Keywords:** UV filter, Human biomonitoring, Metabolism, Phase II conjugates, Urine, Plasma

## Abstract

The chemical UV filter 2-ethylhexyl salicylate (EHS) is used in various personal-care products. The dermal and oral metabolism of EHS have already been targeted by different studies. However, toxicokinetic data after a single dermal exposure to EHS was missing. In our study, three volunteers were dermally exposed to a commercial EHS-containing sunscreen for 9 h with an application dose of 2 mg sunscreen per cm^2^ body surface area. The exposure was performed indoors, and sunscreen was applied on about 75% of the total skin area. Complete urine voids were collected over 72 h and eight blood samples were drawn from each subject. Urine samples were analyzed for EHS and seven known metabolites (5OH-EHS, 4OH-EHS, 2OH-EHS, 6OH-EHS, 4oxo-EHS, 5oxo-EHS, and 5cx-EPS) by online-SPE UPLC MS/MS. The peaks of urinary elimination occurred 10–11 h after application. The elimination half-lives (Phase 1) were between 6.6 and 9.7 h. The dominant urinary biomarkers were EHS itself, followed by 5OH-EHS, 5cx-EPS, 5oxo-EHS, and 4OH-EHS. 2OH-EHS, 6OH-EHS, and 4oxo-EHS were detected only in minor amounts. An enhanced analysis of conjugation species revealed marginal amounts of unconjugated metabolites and up to 40% share of sulfate conjugates for 5OH-EHS, 5oxo-EHS, and 5cx-EPS. The results demonstrated a delayed systemic resorption of EHS via the dermal route. Despite an extensive metabolism, the parent compound occurred as main urinary parameter. The delayed dermal resorption as well as the slow elimination of EHS indicate an accumulation up to toxicological relevant doses during daily repeated dermal application to large skin areas.

**Supplementary Information:**

The online version contains supplementary material available at 10.1007/s00204-024-03827-x.

## Introduction

Sunscreens as well as certain cosmetics and personal-care products contain UV filters whose purpose is to absorb UV radiation to protect skin and hair (Rai et al. 2012; Palm and O'Donoghue 2007; Uter et al. 2014). In total, 29 organic and inorganic substances are approved as UV filters as ingredients in cosmetic products by the European Union (European Union 2021). A prominent UV filter is 2-ethylhexyl salicylate (EHS), which is allowed up to a content of 5% in cosmetic products (European Union 2009). The recommendation to wear sunscreen and the resulting widespread use leads to UV filters being ubiquitous substances, such as in aquatic ecosystems and wastewater-treatment plants (Apel et al. 2018; Cunha et al. 2018; Mitchelmore et al. 2019). The distribution and inevitable exposure to UV filters raises the question of whether there are indications of UV filters having a negative effect on the environment and human health (Huang et al. 2021). Concerning EHS, its toxicological relevance in humans is mainly based on its potential to cause allergic skin reactions in some cases (Shaw 2006; Singh and Beck 2007). In in vitro studies, there is weak endocrine disrupting potential assigned to EHS (Jiménez-Díaz et al. 2013; Kunz and Fent 2006; Morohoshi et al. 2005). Moreover, one study indicates that EHS impairs sperm function (Rehfeld et al. 2018).

The first toxicokinetic data from oral or dermal exposure to EHS are available in the literature (Bury et al. 2019a, 2019b; Hiller et al. 2019). Bury et al. described toxicokinetic data after an oral exposure to EHS, considering the urinary excretion of the metabolites 2-ethyl-5-hydroxyhexylsalicylic acid (5OH-EHS), 2-ethyl-5-oxohexylsalicylic acid (5oxo-EHS), and 5-carboxy-2-ethylpentylsalicylic acid (5cx-EPS) (Bury et al. 2019a, 2019b). Data from a real-life dermal exposure scenario were published by Hiller et al. based on the urinary excretion of EHS and the metabolite 5OH-EHS (Hiller et al. 2019). In a reanalysis of this previous study using a novel, more comprehensive biomonitoring method (Kuhlmann et al. 2024a, 2024b), the parent compound and seven metabolites of EHS (5OH-EHS, 5oxo-EHS, 5cx-EPS, 2-ethyl-4-hydroxyhexylsalicylic acid (4OH-EHS), 2-ethyl-4-oxohexylsalicylic acid (4oxo-EHS), 2-ethyl-6-hydroxyhexylsalicylic acid (6OH-EHS) and 2-(1-hydroxy-ethyl)hexylsalicylic acid (2OH-EHS) could be quantified in the urine after dermal exposure (Kuhlmann et al. 2024c). In 2020, Matta et al. published data from 48 volunteers after repeated sunscreen application over the course of several days, resulting in mean EHS plasma concentrations of 4.6–5.8 µg/L (Matta et al. 2020).

Our working group previously developed a method for the determination of EHS and seven metabolites in urine (Kuhlmann et al. 2024b). This method was now applied in an exposure study. Since the dermal application of sunscreen is the most common exposure scenario, we conducted a controlled dermal-exposure experiment to EHS-containing sunscreen and evaluated the urinary excretion of EHS and its metabolites 2OH-EHS, 4OH-EHS, 5OH-EHS, 6OH-EHS, 4oxo-EHS, 5oxo-EHS, and 5cx-EPS to obtain complete toxicokinetic data. Another aspect which deviates from prior studies is that both glucuronide and sulfate conjugates were considered in the overall analysis and shares of each conjugate were determined. No sunscreen was reapplied in order to calculate the toxicokinetic parameters of a pure single-exposure scenario; this is also the main difference to the study conducted by Hiller et al., which mimicked a real life scenario outdoors, including two reapplications (Hiller et al. 2019).

The objective of this study was to explore the fate and excretion kinetics of EHS after single dermal application of EHS-containing sunscreen.

## Materials and methods

### Study design

For the study, a commercially available sunscreen product with an EHS content of 5% (full list of ingredients in the Online Resource).

The goal of the study was to investigate the toxicokinetics of EHS in sunscreen after dermal exposure under controlled conditions. For this purpose, the exposure scenario used the individual amount of sunscreen necessary to cover 75% of the body surface area (BSA) with an application dose of 2 mg sunscreen per cm^2^ BSA. The BSA (in m^2^) was calculated based on the height (in cm) and weight (in kg) of each study participant based on the following formula:

$BSA=0.007184\times {height}^{0.725}\times {weight}^{0.425}$ (Du Bois and Du Bois 1989).

The exposure was performed indoors to eliminate possible influences of sunlight exposure on the dermal absorption of the sunscreen components. Furthermore, the sunscreen was applied only once at the start of the exposure without any reapplication.

Three volunteers took part in the study, one male and two female (age 23–57). The applied sunscreen amount varied from 24.8 to 31.1 g, resulting in exposure to 1.24–1.55 g EHS. Individual subject characteristics and application amount are summarized in the Online Resource Table S1.

The participants were advised not to use personal-care products containing organic UV filters in the week prior to the exposure and during the sampling period. On the exposure day, the sunscreen was primarily applied by the participants themselves, who wore conventional bathing suits. Body regions covered by clothing as well as the scalp, hands, soles of the feet, the area around the mouth, and the crooks of both arms (to counteract contaminations in blood sampling) were omitted, resulting in exposure of roughly 75% of the individual BSA (as in Matta et al. 2020). The study personnel assisted with application on hard-to-reach body regions—like the back—using a plastic spatula, which was thoroughly wiped on the skin of the participants afterwards. It was therefore ensured, that all designated sunscreen was distributed on the volunteers without any losses. After 9 h, the study participants showered to terminate the exposure.

Regarding sampling, one urine and one blood sample were acquired before the start of exposure to determine background levels. Complete urine samples were collected hourly for the first 12 h, followed by sampling with every necessary urination afterwards. Complete urine voids were collected in 100-mL or 500-mL polypropylene containers until 72 h after sunscreen application. The urine voids were weighted, and creatinine was determined according to Jaffe’s method (Larsen 1972). Blood samples were drawn after 3, 6, 9, 12, 33, and 48 h. The blood samples were centrifuged shortly after sampling, and the plasma was isolated. All samples were stored at − 20 °C.

The ethics committee of the Friedrich-Alexander-Universität Erlangen–Nürnberg approved the conduction of the study (Reg. No. 22–142-B, 25.05.2022). All participants gave their written and informed consent for participation in the study and for the collection of blood and urine samples prior to inclusion in the study.

### Chemicals and instrumentation

For sample analysis, we used an LC–MS/MS system from Waters GmbH (Eschborn, Germany) with online sample clean-up and enrichment. Further information on the analytical method can be found in the Online Resource as well as in the corresponding method publication (Kuhlmann et al. 2024b). Further materials used for the optimization of the plasma method as well as information on the standard substances are summarized in the Online Resource.

### Analytical method for the determination of EHS and metabolites in urine

EHS and its metabolites 5OH-EHS, 4OH-EHS, 2OH-EHS, 6OH-EHS, 4oxo-EHS, 5oxo-EHS, and 5cx-EPS were quantified in urine after enzymatic hydrolysis of the glucuronide and sulfate conjugates using the validated and published LC–MS/MS method by our working group (Kuhlmann et al. 2024b). Limits of detection (LOD) and quantification (LOQ) are summarized in the Online Resource Table S2.

To determine the shares of each conjugate in the urine samples, the sample preparation was modified. The samples were either prepared with no enzyme, with the addition of either sulfatase or glucuronidase, or with the standard sample preparation using both enzymes. All samples were then incubated and further treated according to the standard sample preparation.

### Determination of EHS and its metabolites in human plasma

Plasma samples were collected in the framework of the dermal exposure experiment to acquire knowledge on the systemic levels of EHS and metabolites. Therefore, an analytical method for the determination of EHS and its metabolites in human plasma was developed, which is described in detail in the Online Resource (Chapter S6). During the method validation, issues in robustness were observed regarding the quantification of several parameters after the storage of plasma samples at − 20 °C. We concluded that said issues are most likely caused by a remaining activity of esterases in human plasma, resulting in an ester cleavage of the salicylate esters. These findings are described in detail in the Online Resource. Based on these findings, we concluded that a reliable determination of the parameters of interest in the collected and stored plasma samples is not possible. Hence, the obtained plasma samples from the dermal exposure experiment were not analyzed.

### Data evaluation

The analysis results were obtained in µg/L. Samples with concentrations below the LOD were included into the kinetic calculations as half the LOD. The following urinary kinetic parameters were calculated:

Renal excretion rates (*R*_E_, µg/h) were calculated for each analyte at a certain point in time using the equation:

$${R}_{\text{E}}=\frac{{c}_{i} \times {v}_{i}}{{t}_{i}-{t}_{i-1}}$$

The concentration *c*_*i*_ (in µg/L) is the concentration of the considered analyte in the urine sample *i*, *v*_*i*_ (in L) is the volume of the urine sample, *t*_*i*_ (in h) is the time since the start of the exposure, and *t*_*i-1*_ (in h) is the time elapsed since the previous sample.

The renal excretion kinetics for each analyte were plotted as the renal excretion rates *R*_E_ against the midpoint of the respective sampling period (*t*_*i,*m_, in h):

$${t}_{i,\text{m}}={t}_{i-1}+\frac{{t}_{i}- {t}_{i-1}}{2}$$

For each participant and analyte, excretion curves were prepared separately by plotting the excretion rates against the corresponding average time of the sampling period. In order to obtain mean excretion curves, the closest sampling time spots and the corresponding renal excretion rates of all study participants were averaged. The mean excretion curves were then ln-transformed and the slopes *k*_el_ (elimination rate constant) were determined. Based on the elimination rate constant, the elimination half-lives (*t*_1/2_) were calculated:

$${t}_{1/2}=\frac{\text{ln}(2)}{|{k}_{\text{el}}|}$$

To calculate the cumulative excreted amount of each analyte and participant (in µmol), the molar excreted amounts of each analyte are summed using the molar mass of the respective analyte (*M*, in µg/µmol):

$${\sum }_{i=0}^{n}\frac{{c}_{i}\times {v}_{i}}{M}$$

To express the total excretion of EHS and its metabolites, the urinary excretion fractions (*F*_UE_) as equivalents of the applied EHS dose (as percentages) were calculated after 24 h, 48 h, and 72 h:

$${F}_{\text{UE}}=\frac{{CE}_{i}}{{M}_{\text{D}}}\times 100$$

*CE*_*i*_ represents the amount of the respective analyte excreted after 24 h, 48 h, and 72 h (in µmol). *M*_D_ is the dermally applied amount of EHS (in µmol).

The shares of glucuronide and sulfate conjugates were assessed by calculating the cumulative excreted amount after hydrolysis with either glucuronidase or sulfatase and calculating the shares by dividing the respective cumulative excreted amount by the sum of the cumulative excretion after glucuronide and sulfate hydrolysis. To assess unconjugated shares, the cumulative excreted amount was also calculated based on concentrations following omitted conjugate hydrolysis. For EHS, the cumulative excreted amount after hydrolysis without glucuronidase could not be calculated since a glucuronide is used as an internal standard. The assessment of relevant sulfate conjugation and unconjugated shares was therefore performed by evaluating the peak areas as well as comparing the cumulative amounts after hydrolysis with both enzymes and with only glucuronidase.

Metabolites were correlated by plotting the respective concentrations (in µg/L) against each other and calculating the Pearson correlation coefficients (*r*).

The data was processed using Microsoft Excel®. Origin (2019)® was used for curve-fitting.

## Results and discussion

### Toxicokinetics of EHS and metabolites in urine

The urine samples collected before the start of the exposure had concentrations above the LOQ for all analytes except 6OH-EHS and 4oxo-EHS. However, the concentrations were always below 1 µg/L, which is very low compared to the levels after exposure. Even though the participants were advised not to use any EHS-containing products, the widespread use and contamination of EHS in products used or food consumed by the general population may lead to a low level of unintentional background exposure. In the urine samples collected after dermal exposure to EHS-containing sunscreen, all analytes could be detected, albeit in varying shares. The renal excretion kinetics are summarized in Table 1.

**Table 1:** Renal excretion kinetics of all analytes after dermal exposure to EHS-containing sunscreen (*n* = 3; mean ± SD)

	EHS	5OH-EHS	4OH-EHS	2OH-EHS	6OH-EHS	4oxo-EHS	5oxo-EHS	5cx-EPS
*t*_max_ [h]	11.1 ± 0.4	11.1 ± 0.4	10.7 ± 0.6	11.1 ± 0.4	10.8 ± 0.3	10.2 ± 1.3	11.1 ± 0.4	11.1 ± 0.4
*c*_max_ [µg/g creatinine]	115.1 ± 47.6	46.7 ± 15.8	31.7 ± 11.2	5.2 ± 3.4	0.5 ± 0.4	2.3 ± 1.0	36.7 ± 23.7	43.7 ± 13.8
*RE*_max_ [µg/h]	6.69 ± 1.04	2.74 ± 0.35	1.72 ± 0.13	0.26 ± 0.10	0.02 ± 0.02	0.13 ± 0.03	2.05 ± 0.83	3.26 ± 1.99
*t*_1/2_ Phase 1 [h]	6.6	7.9	7.3	8.4	*	7.8	9.7	9.4
*t*_1/2_ Phase 2 [h]	19.0	25.8	28.0	21.6	-**	17.1	45.6	36.0
Cumulative excreted amount [µmol]	0.411 ± 0.072	0.181 ± 0.034	0.109 ± 0.010	0.018 ± 0.007	0.001 ± 0.0003	0.007 ± 0.001	0.128 ± 0.059	0.186 ± 0.074
F_UE_ after 24 h [%]	0.00582 ± 0.00145	0.00248 ± 0.00059	0.00154 ± 0.00030	0.00026 ± 0.00012	0.00001 ± 0.00001	0.00010 ± 0.00003	0.00179 ± 0.00100	0.00222 ± 0.00075
F_UE_ after 48 h [%]	0.00711 ± 0.00193	0.00310 ± 0.00078	0.00189 ± 0.00039	0.00032 ± 0.00015	0.00001 ± 0.00001	0.00013 ± 0.00003	0.00226 ± 0.00125	0.00305 ± 0.00100
F_UE_ after 72 h [%]	0.00756 ± 0.00200	0.00330 ± 0.00082	0.00199 ± 0.00037	0.00034 ± 0.00016	0.00001 ± 0.00001	0.00013 ± 0.00003	0.00240 ± 0.00131	0.00328 ± 0.00105

*RE*_max_ maximum renal excretion rate, *t*_max_ time point of maximum renal excretion rate, *c*_max_ max. observed concentration, *t*_1/2_ elimination half-life, F_UE_ urinary excretion fraction. *Low renal excretion rate resulted in a theoretical value of 36.1 h. **Because of the low renal excretion rate, no biphasic excretion kinetic could be identified

The kinetics of all monitored parameters were very similar, at least in the pre-maximum phase and the first elimination phase. Maximum renal excretion rates were reached 10–11 h after sunscreen application. EHS shows the highest mean maximum urinary excretion rate with 6.7 µg/h, followed by 5cx-EPS, 5OH-EHS, and 5oxo-EHS, all with excretion rates above 2 µg/h. Lower excretion rates below 2 µg/h were found for 4OH-EHS, 2OH-EHS, 6OH-EHS, and 4oxo-EHS.

**Fig. 1:**
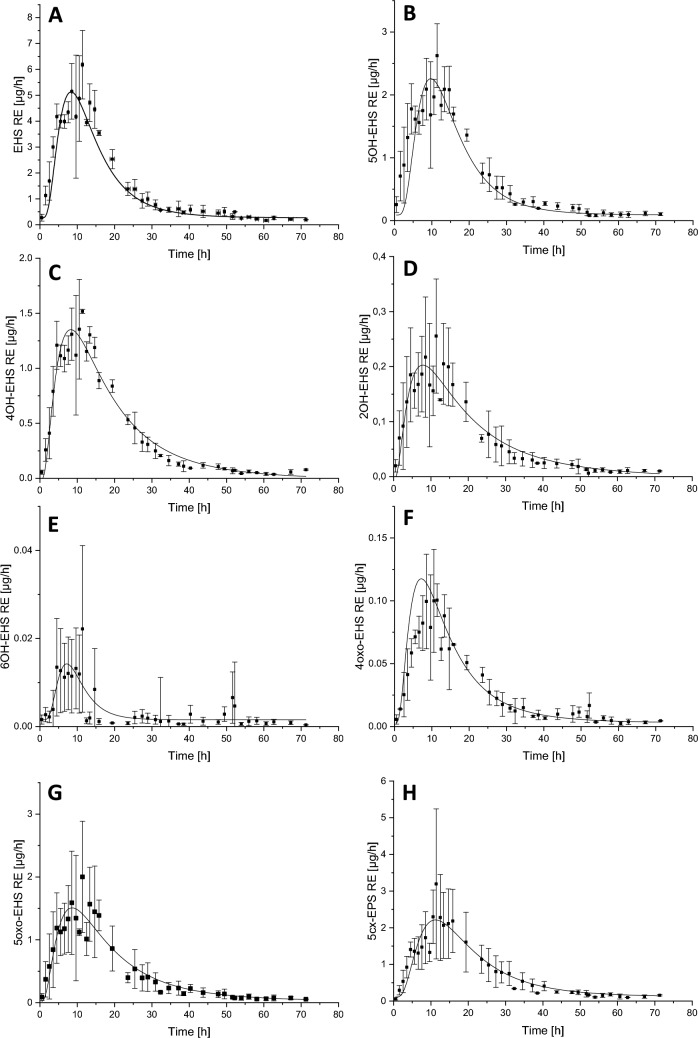
Mean urinary excretion rates of **A** EHS, **B** 5OH-EHS, **C** 4OH-EHS, **D** 2OH-EHS, **E** 6OH-EHS, **F** 4oxo-EHS, **G** 5oxo-EHS, and **H** 5cx-EPS after dermal exposure to EHS-containing sunscreen with log-normal fit (*n* = 3)

In Fig. 1, the temporal progression of the urinary excretion of all analytes is displayed. For the calculation of the excretion half-lives, the mean renal excretion rates were ln-transformed (graphs are displayed in the Online Resource, Fig. S1), whose courses indicate clearly a multi-phase excretion kinetics and breakpoints between the phases. Elimination half-lives were therefore calculated separately for phase 1 and phase 2, based on evaluation of the thereof observed time frames. Biphasic elimination was observed with all analytes except 6OH-EHS, where, due to low renal excretion, a clear differentiation of the elimination phases was not possible.

The urinary excretion fractions were rather low, with only 0.00001–0.0071% of the dermally applied dose recovered as EHS and its metabolites in urine after 42 h. Similar urinary excretion fractions were found in the previous volunteer study under real-life conditions (Hiller et al. 2019; Kuhlmann et al. 2024c). Bury et al. found urinary excretion fractions of 0.28%, 0.23%, and 0.11% for 5OH-EHS, 5cx-EPS, and 5oxo-EHS, respectively, for the same period after oral administration, which is higher by a factor of about 100 (Bury et al. 2019b). Thus, the low urinary excretion fraction may be explained by the low dermal resorption rate rather than by restricted metabolism and elimination. In vitro data are available, positing around 0.5% percutaneous absorption of EHS when applied in an emulsion vehicle (Scientific Committee on Cosmetology 2000; Treffel and Gabard 1996; Walters et al. 1997).

EHS itself makes up the highest share of the urinary excreted analytes with 40% ± 4%, followed by 5cx-EPS with 18% ± 7%, 5OH-EHS with 17% ± 1%, 5oxo-EHS with 12% ± 4%, and 4OH-EHS with 11% ± 2%. The shares of 2OH-EHS, 6OH-EHS, and 4oxo-EHS were all below 2%. Two of the prominent analytes 5OH-EHS and 5cx-EPS, are categorized as biomarkers for EHS by the German Federal Environment Agency and were applied in a study on the time course of EHS exposure in the German population (Bury et al. 2023; HBM Commission 2023). The acquired data can be compared to the studies by Hiller et al. and Bury et al. (Bury et al. 2019a, 2019b; Hiller et al. 2019). The study by Hiller et al. covered a real-life exposure scenario with 20 volunteers, sunscreen re-application, and an outside venue. EHS and 5OH-EHS were assessed as analytes (Hiller et al. 2019). The maximum concentrations for both analytes were roughly twice as high than those quantified here. This finding is in good correlation with the exposure scenario, since Hiller et al. reapplied sunscreen twice with 50% of the initial amount of sunscreen each and calculated the designated sunscreen amount for 100% of the BSA. Therefore, the dermally applied dose in this former study was more than doubled in comparison with the present study. Regarding the excretion shares, our data is in line with Hiller et al. with EHS being excreted to a larger extent than 5OH-EHS. Urinary excretion within 24 h in comparison to the initially applied dose lies within the same order of magnitude as well (Hiller et al. 2019). Bury et al. assessed the toxicokinetics of EHS after oral exposure with three volunteers (included analytes: 5OH-EHS, 5oxo-EHS, and 5cx-EPS) (Bury et al. 2019b). After oral dosage, the elimination half-life of Phase 1 is shorter by several hours in comparison to dermal exposure. The excretion kinetics after dermal exposure are therefore rather slow in comparison to oral exposure. The slow dermal resorption can cause a shift in the time-point of maximum RE but also may affect the excretion kinetics by a hindered uptake of the compound. Moreover, the lack of a first-pass effect may explain deviations in the metabolite distribution after oral and dermal exposure. Bury et al. also conducted an initial dermal-exposure experiment with EHS-containing sunscreen with one volunteer. Their data is in line with our findings that 5OH-EHS, 5cx-EPS, and 5oxo-EHS can be quantified in urine after dermal exposure to EHS. Furthermore, the longer elimination half-lives in comparison to oral exposure were described (Bury et al. 2019a, 2019b). The overall slow elimination after dermal exposure can be derived from the cumulative excretion of EHS-equivalents after 24 h in comparison to 72 h. Assuming a complete excretion after 72 h, the share of EHS-equivalents excreted after 24 h is 25–26%. An exposure on consecutive days could therefore lead to accumulative effects.

**Fig. 2:**
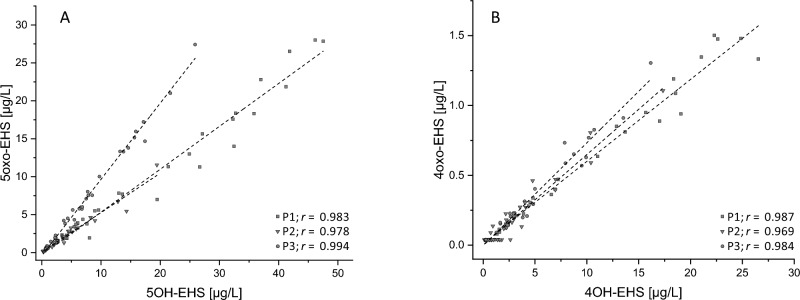
Correlations of metabolite concentrations after controlled dermal exposure to EHS-containing sunscreen. **A** 5oxo-EHS vs. 5OH-EHS. **B** 4oxo-EHS vs. 4OH-EHS

**Fig. 3:**
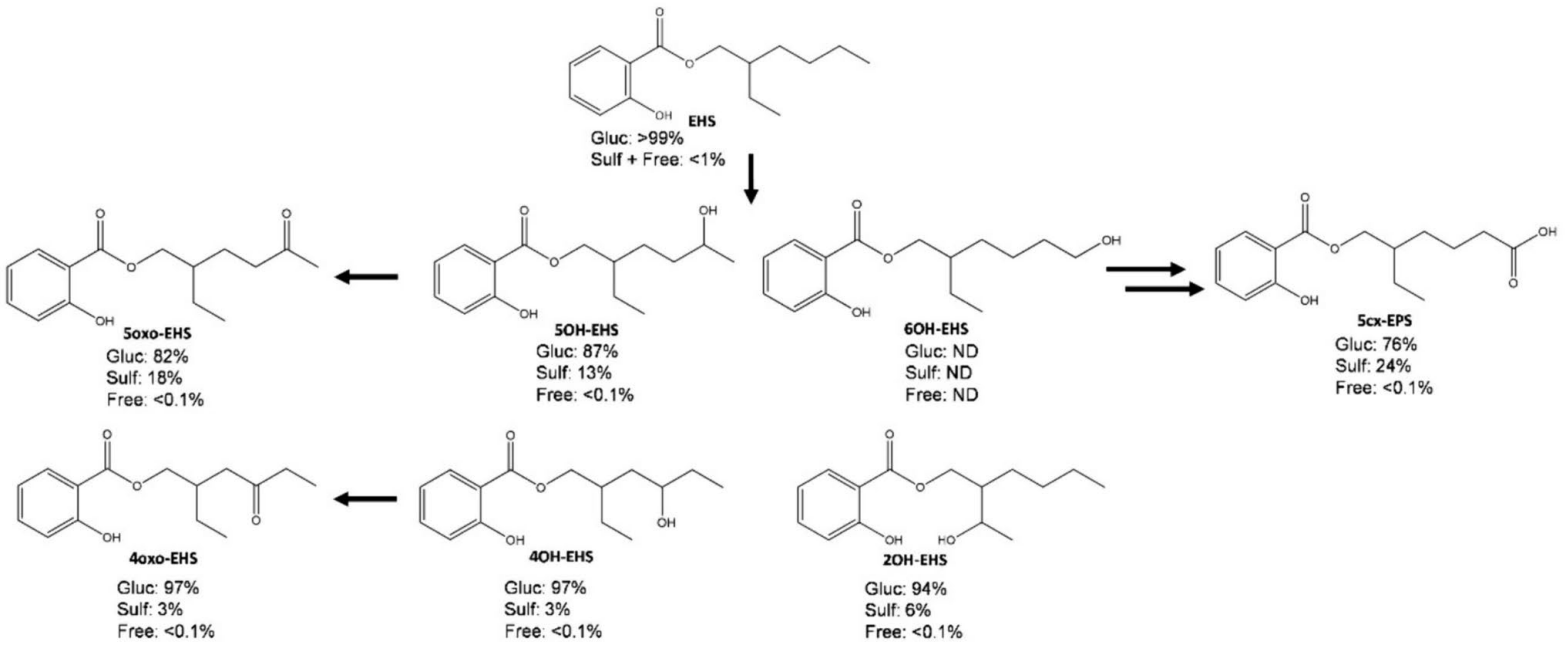
Metabolic scheme of EHS and oxidative metabolites including the mean shares of glucuronide (Gluc) and sulfate (Sulf) conjugations determined in the three study participants. *ND* = not determined

The relevant oxidation positions in the ethylhexyl chain were presented in our publication on new metabolites found in real-life dermal exposure samples (Kuhlmann et al. 2024c), namely, oxidation in ω (5cx-EPS, 6OH-EHS), ω-1 (5OH-EHS, 5oxo-EHS), and ω-2 (4OH-EHS, 4oxo-EHS) could be confirmed in the present study. Correlations of the metabolites oxidated in the ω-1 and ω-2 positions, respectively, are displayed in Fig. 2. In addition, an overall metabolic scheme including the chemical structures is displayed in Fig. 3.

Due to the high share of 6OH-EHS concentrations below the LOQ, a reasonable correlation with 5cx-EPS to represent oxidation in the ω position was not feasible. The metabolites oxidated in the ω-1 and ω-2 positions each show a very high correlation with the Pearson correlation coefficient above 0.96 in all cases, thus confirming the coherence of the respective oxidation pathway.

The correlation between 5oxo-EHS and 5OH-EHS showed distinct interindividual variance, as the slope for data of one volunteer were about twice as high as the slopes for the two other volunteers (Fig. 2A). In contrast, an interindividual variance regarding the 4oxo-EHS and 4OH-EHS correlation lines were not found (Fig. 2B). The different ratios between oxo-EHS and OH-EHS for the ω-1 and ω-2 position of the hexyl chain imply a steric hindrance in the active site of the alcohol dehydrogenase (Kuhlmann et al. 2024c). It can be assumed that a similar effect may be responsible for interindividual variation of the ratios between 5oxo-EHS and 5OH-EHS considering the involvement of different isoezymes. Intensity and coverage of the interindividual variance needs further investigations.

### Shares of glucuronide conjugates, sulfate conjugates and unconjugated species

Unconjugated shares of the oxidative metabolites in the cumulative excreted amounts were overall below 0.1%. The evaluation of EHS peak areas without conjugate hydrolysis also showed no significant distinction from reagent blank measurements. Therefore, EHS and the metabolites targeted in this study are predominantly excreted in conjugated forms, which is in line with the findings of Bury et al. regarding the conjugation of 5OH-EHS, 5oxo-EHS, and 5cx-EPS after oral application as well as previous studies from our working group on the stability of unconjugated EHS (Bury et al. 2019b; Kuhlmann et al. 2024a).

In Table 2, the glucuronide and sulfate conjugate shares of the oxidative metabolites, categorized by study participant, are summarized. Due to overall low concentrations or concentrations below the LOQ, 6OH-EHS shares could not be evaluated. No significant sulfate shares in the conjugation of EHS and predominant conjugation to glucuronic acid were found in the evaluation of the EHS peak areas after hydrolysis with only sulfatase as well as comparison of the EHS concentrations after hydrolysis with only glucuronidase with concentrations after hydrolysis with both enzymes. In Fig. 3, the metabolic scheme including the mean conjugate shares is displayed.

**Table 2:** Shares of glucuronide and sulfate conjugates after dermal exposure to EHS-containing sunscreen

	Study participant	5OH-EHS	4OH-EHS	2OH-EHS	4oxo-EHS	5oxo-EHS	5cx-EPS
*glucuronide share [%]*	1	77	94	88	92	65	65
	2	96	100	100	100	92	86
	3	88	98	93	100	89	76
*sulfate share [%]*	1	23	6	12	8	35	35
	2	4	–	–	–	8	14
	3	12	2	7	–	11	24

The data regarding the sulfate and glucuronide shares of oxidative EHS metabolites support the previous findings that there are considerable amounts of sulfate conjugates present for 5OH-EHS, 5oxo-EHS, and 5cx-EPS (Kuhlmann et al. 2024b). Other phenolic compounds also show significant conjugation to sulfate, for example parabens, bisphenols, and polychlorinated phenols (Denghel and Göen 2023; Dhakal et al. 2018). Interestingly, the share of sulfate conjugates shows high variability between the study participants. A trend regarding age or gender cannot be derived due to the small number of participants. Despite the high interindividual differences in the sulfate shares, the individual tendency to higher or lower shares stayed consistent across all EHS metabolites; Participant 2 always exhibited the lowest and Participant 1 always the highest values. This finding indicates individually varying activities of sulfo- (SULT) and UDP-glucuronosyltransferases (UGT) that lead to generally higher or lower sulfate shares on the individual level. Interindividual UGT and SULT variability is demonstrated in several studies (Achour et al. 2014; Braver-Sewradj et al. 2018; Macià et al. 2023; Riches et al. 2009). However, the urine samples of participant #2 were frequently higher diluted regarding the creatinine content, which may limit the robustness of results. The presented data again highlights the importance of including sulfatase in the hydrolysis step to correctly determine the excretion of the prominent biomarkers 5OH-EHS, 5oxo-EHS, and 5cx-EPS.

## Conclusions

The results of the study describe a clear and plausible toxicokinetic of EHS in humans after single, controlled dermal exposure to the compound. The results confirmed that the urinary excretion of the parent compound represents by far the most prominent parameter for dermal exposure to EHS. This is a remarkable distinction compared to the data found after oral exposure, which did not indicate any renal excretion of EHS in relevant amounts. Furthermore, the study showed extensive oxidative metabolism of EHS after dermal exposure by the identification of seven metabolites of different oxidative stages. The toxicokinetics of EHS and its metabolites were very similar during the resorption phase as well as in the first phase of elimination. The maximum renal excretion rate at 10–11 h after the dermal application demonstrated a retarded uptake of the compound, which is in line with the dermal resorption kinetics of other rather lipophilic compounds. The oxidative metabolites as well as EHS itself were excreted efficiently via the urine after conjugation to both glucuronic acid and sulfate. A specific analysis revealed a predominant conjugation as glucuronide, but also considerable amounts of sulfate conjugation for 5OH-EHS, 5oxo-EHS and 5cx-EPS. This finding highlights the importance of including sulfate conjugates in the assessment strategy, especially as significant interindividual differences in the Phase II metabolism were found.

The delayed dermal resorption as well as the slow elimination of EHS indicate an accumulation of the body burden up to toxicologically relevant doses during daily repeated dermal application to large skin areas. As a result, a benefit–risk consideration is recommended in appropriate application scenarios for consumers as well as in the workplace.

## Supplementary Information

Below is the link to the electronic supplementary material.

Supplementary file1 (PDF 895 KB)

## Data Availability

Data are stored under controlled access at the Institute and Outpatient Clinic of Occupational, Social, and Environmental Medicine at Friedrich-Alexander-Universität Erlangen-Nürnberg. Anonymized raw data, not otherwise included in the article or online supplement, are available from the corresponding author upon reasonable request.

## Abbreviations

EHS: 2-Ethylhexyl salicylate
EHS-GlcA: 2-Ethylhexyl salicylate glucuronide
5OH-EHS: 2‑Ethyl‑5‑hydroxyhexyl 2‑hydroxybenzoate
2OH-EHS: 2‑Ethyl‑2‑hydroxyhexyl 2‑hydroxybenzoate
4OH-EHS: 2‑Ethyl‑4‑hydroxyhexyl 2‑hydroxybenzoate
6OH-EHS: 2‑Ethyl‑6‑hydroxyhexyl 2‑hydroxybenzoate
4oxo-EHS: 2‑Ethyl‑4‑oxohexyl 2‑hydroxybenzoate
5oxo-EHS: 2‑Ethyl‑5‑oxohexyl 2‑hydroxybenzoate
5cx-EPS: 5‑(((2‑Hydroxybenzoyl)oxy)methyl)heptanoic acid
SULT: Sulfotransferase
UGT: UDP-glucuronosyltransferase
