# Supplementary material for: Toxicokinetics of 2-ethylhexyl salicylate (EHS) and its seven metabolites in humans after controlled single dermal exposure to EHS
Source: Arch Toxicol. 2024 Aug 12;98(10):3259–68. doi: 10.1007/s00204-024-03827-x (PMC11402844; doi:10.1007/s00204-024-03827-x)
Supplement: Supplementary file 1 — Supplementary file1 (PDF 895 KB) [file 204_2024_3827_MOESM1_ESM.pdf]

Supplementary material for:

Toxicokinetics of 2-ethylhexyl salicylate (EHS) and its seven metabolites in humans after controlled single dermal exposure to EHS

In: Archives of Toxicology

Laura Kuhlmann, Thomas Göen, Julia Hiller

Institute and Outpatient Clinic of Occupational, Social, and Environmental Medicine, Friedrich-Alexander-Universität Erlangen-Nürnberg, Henkestr. 9–11, 91054 Erlangen, Germany

Corresponding author: Dr. med. Julia Hiller, Tel.: +49 9131 8526116

Email: Julia.hiller@fau.de

S1. Sunscreen used for exposure

German drugstore store brand ‘DM Sundance 30 hoch’. An analysis of the Bavarian Office for Health and Food Safety in 2021 stated following UV-filter content for the used charge: Butyl Methoxydibenzoylmethane (Avobenzone) 2,1 %, Ethylhexyl Salicylate 5,0 %, Octocrylene 7,0 %, Phenylbenzimidazole Sulfonic Acid 0,5 %, Tris-Biphenyl Triazine (nano). The complete ingredients are listed below:

Aqua; Octocrylene; Glycerine; Alcohol Denat.; Ethylhexyl Salicylate; Butyl Methoxydibenzoylmethane; Coco Caprylate/Caprate; C12-C15 Alkyl Benzoate; Undecane; Tocopheryl Acetate; Tricontanyl PVP; Tris-Diphenyl Triazine (nano); VP/Hexadecene Copolymer; Tridecane; Phenylbenzimidazole Sulfonic Acid; Parfum; Acrylates/C10-30 Alkyl Acrylate Crosspolymer; Caprylyl Glycol; Sodium Hydroxide; Carnosine; Xanthan Gum; Decyl Glucoside; Carbomer; Disodium EDTA; Maltodextrin; Limonene; Alpha-Isomethyl Ionone; Caprylhydroxamic Acid; Butylene Glycol; Disodium Phosphate; Camellia Sinensis Leaf Extract; Benzyl Alcohol; Tocopherol; Helianthus Annuus Seed Oil

S2. Detailed participant exposure data

Table S 1: Characteristics and applied sunscreen amount for each study participant.

	Participant 1	Participant 2	Participant 3
Gender	Male	Female	Female
Age [years]	57	29	23
Body surface area [m^2]	2.07	1.84	1.68
Sunscreen applied [g]	31.05	27.53	24.82
EHS applied [g] (calculated from content percentage)	1.55	1.38	1.24

S3. Information on the LC-MS/MS System

The LC-MS/MS was consisting of a Waters ACQUITY® UPLC H-Class system with two pumps, an autosampler with temperature control (Waters ACQ H-Class FTN-H Plus), and a column manager (ACQUITY® UHPLC CM-A). For enrichment, a C8 phase (XBridge® BEH C8 Direct Connect HP 30 mm x 2.1 mm, 10 µm from Waters, Eschborn, Germany) was used. The separation was performed on a reversed-phase column (ACQUITY® UPLC HSS T3, 1.8 µm, 2.1 mm x 150 mm with the corresponding pre-column, both from Waters, Eschborn, Germany). The LC system was attached to the Waters Xevo® TQ-XS mass spectrometer including an electrospray source operating in negative ionization mode. MS-parameters are set to 1.5 kV capillary voltage, 150 °C source temperature, 500 °C desolvation temperature and 1000 L/h flow. Nitrogen was used as desolvation gas and argon as collision gas.

S4. Limits of detection and quantification

Table S 2: Limits of detection (LOD) and quantification (LOQ) in urine according to the method by Kuhlmann et al. (2024).

	EHS	5OH-EHS	4OH-EHS	2OH-EHS	6OH-EHS	4oxo-EHS	5oxo-EHS	5cx-EPS
LOD [ng/L]	65	9	6	8	6	24	17	16
LOQ [ng/L]	210	31	21	29	22	79	59	55

S5. Ln-transformed renal excretion rates

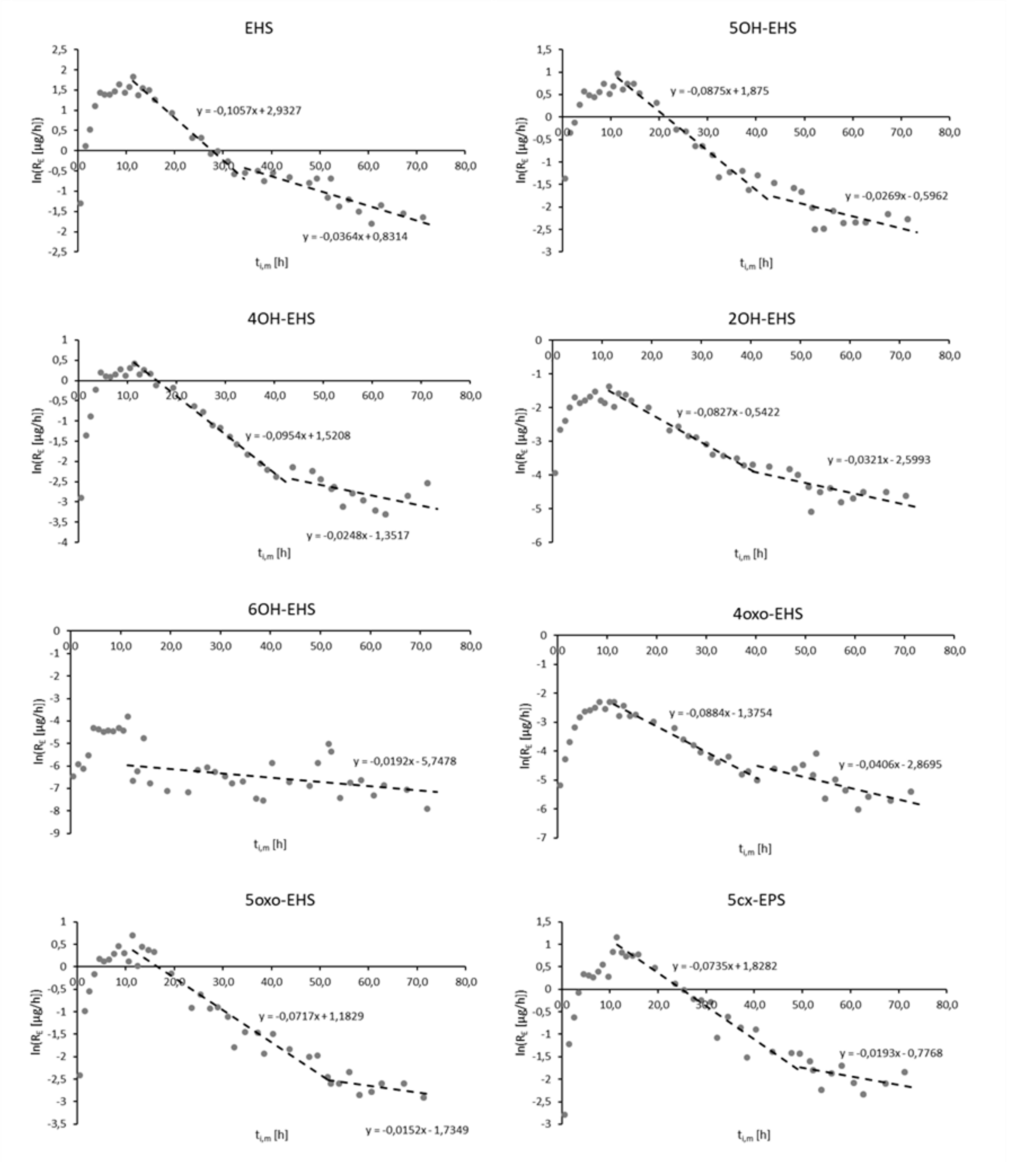

Figure S 1: ln-transformed mean renal excretion rates of all analytes for the elimination half-life evaluation.

S6. Plasma method

S6.1. Standard substances

2-ethylhexyl salicylate (EHS), salicylic acid and D4-salicylic acid were purchased from Merck (Darmstadt, Germany) with a purity of at least 99%. The following reference substances and internal standards were all synthesized at the Max Planck Institute for Biophysical Chemistry, Facility for Synthetic Organic Chemistry (Göttingen, Germany): 2-[((2-ethylhexyl)oxy)carbonyl]phenol-β-D-glucuronic acid (EHS-GlcA), 2-[((2-ethyl¬hexyl)¬oxy)-carbonyl]-phenol-3,4,5,6-D_4-β-D-glucuronic acid triethylammonium salt (D_4-EHS-GlcA), 2-ethyl-5-hydroxy¬hexyl 2-hydroxybenzoate (5OH-EHS), 2-ethyl-5-hydroxyhexyl 2-hydroxybenzoate-3,4,5,6-D_4 (D_4-5OH-EHS), 2-ethyl-4-hydroxyhexyl 2-hydroxybenzoate (4OH-EHS), 2-ethyl-4-hydroxyhexyl 2-hydroxybenzoate-3,4,5,6-D_4 (D_4-4OH-EHS), 2-(1-hydroxy-ethyl)hexyl 2-hydroxy¬benzoate (2OH-EHS), 2-(1-hydroxy-ethyl)hexyl 2-hydroxy¬benzoate-3,4,5,6-D_4 (D_4-2OH-EHS), 2-ethyl-6-hydroxyhexyl 2-hydroxybenzoate (6OH-EHS), 2-ethyl-6-hydroxyhexyl 2-hydroxybenzoate-3,4,5,6-D_4 (D_4-6OH-EHS), 2-ethyl-5-oxohexyl 2-hydroxybenzoate (5oxo-EHS), 2-ethyl-5-oxohexyl 2-hydroxybenzoate-3,4,5,6-D_4 (D_4-5oxo-EHS), 2-ethyl-4-oxohexyl 2-hydroxybenzoate (4oxo-EHS), 2-ethyl-4-oxohexyl 2-hydroxybenzoate-3,4,5,6-D_4 (D_4-4oxo-EHS), 4-(((2-hydroxybenzoyl)oxy)methyl)hexanoic acid (5cx-EPS) and 4-(((2-hydroxybenzoyl-3,4,5,6-D_4)oxy)methyl)hexanoic acid (D_4-5cx-EPS). The chemical purity was in all cases at least 95%. The isotopic purity of the internal standards was tested by LC-MS/MS and the standards contained no measureable unlabeled compound.

S6.2. Chemicals and materials for sample preparation and LC-MS/MS

Enzymes for deconjugation were β-Glucuronidase (from *E. coli* K12) purchased from Roche Biomedical (Mannheim, Germany) and sulfatase from *Aerobacter aerogenes* Type VI from Merck (Darmstadt, Germany). Ammoniumacetate, formic acid, acetic acid, acetonitrile, water, and methanol (all UPLC grade) were purchased from VWR Chemicals (Darmstadt, Germany). Pooled human plasma for method development was supplied from in.vent Diagnostica GmbH (Henningsdorf, Germany).

S6.3. Development of the analytical method for the determination of EHS and metabolites in human plasma

Based on the work by Klotz et al. to establish a method for the determination of EHS and 5OH-EHS in human plasma, we continued method development with the overall goal of quantifying EHS and seven metabolites after enzymatic hydrolysis in plasma (Klotz et al. 2019). To cover both protein precipitation and conjugate hydrolysis during sample preparation, protein participation had to be conducted before the hydrolysis step. Since the standard substances for EHS and its corresponding glucuronide (EHS-GlcA) were available, the extraction efficiency from plasma for EHS and EHS-GlcA was tested as described below.

In the final procedure, 300 µL plasma was spiked with 10 µL of the internal standard solution (in acetonitrile). Afterwards, 600 µL acetonitrile was added for protein participation. The samples were vortexed for one minute following centrifugation at 3000 rpm (rounds per minute) for 10 minutes. Subsequently, 700 µL of the supernatant were transferred into a fresh vial and evaporated under a nitrogen stream to a residual volume of 100 µL. Finally, 500 µL of the ammonium-acetate buffer (1M,

pH 6.5) and 5 µL of each enzyme (glucuronidase and sulfatase) were added and the samples were incubated for 2 h at 37 °C. After incubation, 20 µL are injected into the LC-MS/MS system. The LC-MS/MS analysis was done with the same method established for the analysis in urine (Kuhlmann et al. 2024). Validation of the analytical method was attempted as well.

S6.4. Testing of the analyte extraction efficiency in human plasma

300 µl of pooled human plasma was spiked separately with glucuronidated EHS and unconjugated EHS standard solutions, resulting in 33 µg/L EHS-equivalents each. The samples were prepared in threefold. All samples were spiked with 5 µl D_4-EHS-GlcA standard solution as internal standard. 600 µl acetonitrile is added to the plasma samples for precipitation. Following the precipitation, the sample preparation is done analogously to the description in the publication. After LC-MS/MS analysis the area as well as the response (analyte peak area divided by the area of the internal standard) of the samples spiked with conjugated and unconjugated EHS were compared by t-test. The p-values for both area ($p = 0.114$) and response ($p = 0.085$) were above 0.05, therefore, no significant differences between the unconjugated and glucuronidated EHS plasma samples could be stated.

S6.5. LC-MS/MS Method for the determination of salicylic acid in human plasma

For the determination of salicylic acid (SA) in human plasma, an LC-MS/MS method with online enrichment and sample clean-up was used. System and columns were the same as described under S2. For the online enrichment and chromatographic separation, an 18-minute gradient of methanol (A) and water with 0.01% acetic acid (B) was used at a flow rate of 0.6 mL/min for enrichment and 0.25 mL/min for chromatographic separation. The program for the enrichment column started isocratic with 1% A until 2.1 minutes, then A was increased to 95% within 1.8 minutes and kept stable for 2.1 minutes. Within 1 minute, A was increased to 100% and was maintained for 3 minutes. Within 1 minute, A was lowered back to the initial value of 1% and the column was conditioned for 7 minutes. The gradient program for the chromatography column was held at 50% A for 5 minutes, then A was increased to 75% within 1 minute and held for 6 minutes. Within 2 minutes A was increased again to 100% and held for 2.5 minutes. Afterwards, A was lowered back to 50% within 0.5 minutes and held stable for 1 minute. Regarding the column switching program, the sample enrichment and clean-up on the enrichment column lasted 2 minutes before the valve switched and the analytes were eluted from the enrichment column in back-flash mode onto the chromatography column. After 2 minutes transfer time, the valve switched back into the initial state. The sample department was cooled to 8 °C and the injection volume was set to 20 µL. As analytes, all analytes besides EHS were included into the MS detection method, these transitions can be found in the method paper by our working group (Kuhlmann et al. 2024). EHS could not be included because including the polar SA and the far less polar EHS in the same online LC method was not possible. For SA, *m/z* 137 was chosen as precursor ion and *m/z* 93 as quantifier as well as *m/z* 65 as qualifier. For D_4-SA, the transition *m/z* 141 to *m/z* 97 was used.

S6.6. Sample preparation for the determination of salicylic acid in plasma

300 µl plasma sample was spiked with 10 µl of internal standard solution (c = 300 µg/L for each internal standard), then 50 µl 1% formic acid was added and samples were mixed by shaking. Afterwards,

600 µl acetonitrile was added for protein precipitation. Samples were vortexed and centrifuged at 3000 rpm (rounds per minute) for 10 minutes. 700 µl supernatant was transferred into fresh vials and 20 µL were injected into the LC-MS/MS system.

S6.7. Results for testing of the analyte cleavage to salicylic acid in plasma

For the experiments regarding the salicylic acid increase in stored plasma samples, we spiked pooled plasma with 40 µg/L analytes (5OH-EHS, 2OH-EHS, 4OH-EHS, 6OH-EHS, 4oxo-EHS, 5oxo-EHS and 5cx-EPS), aliquoted 300 µl samples in amber glass vials and stored them together with 300 µl blank plasma aliquots at –20 °C. For the sample work up, samples were prepared in three-fold together with one freshly spiked (40 µg/L) plasma sample, the addition of enzymes and the hydrolysis step was omitted, since the standard substances were unconjugated. Samples were analyzed after initial preparation, as well as 2, 7 and 14 days of storage. The course of response and area over time for salicylic acid and 5OH-EHS (representing the trend for all covered analytes) is shown in Figure S 2 – S 5.

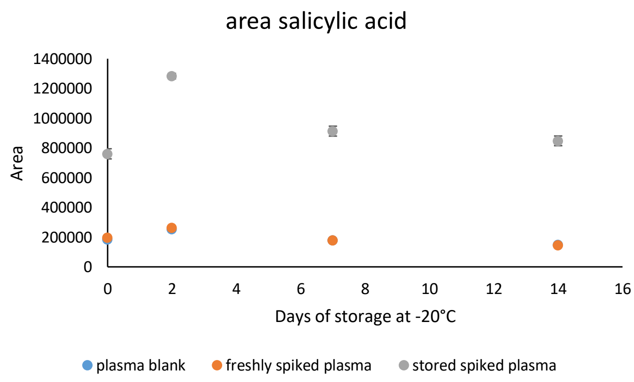

Figure S 2: area of salicylic acid in plasma over the course of 14 days.

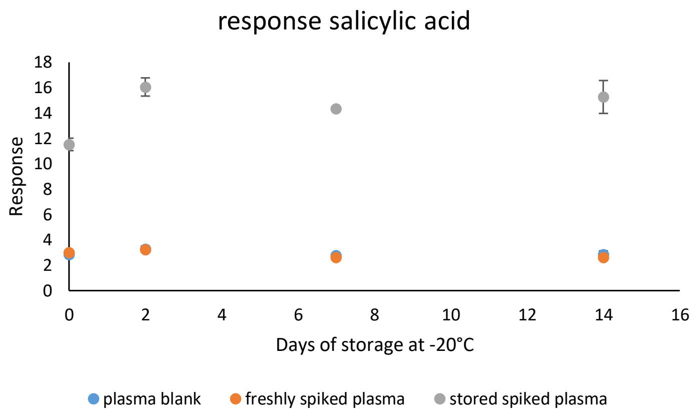

Figure S 3: response of salicylic acid in plasma over the course of 14 days.

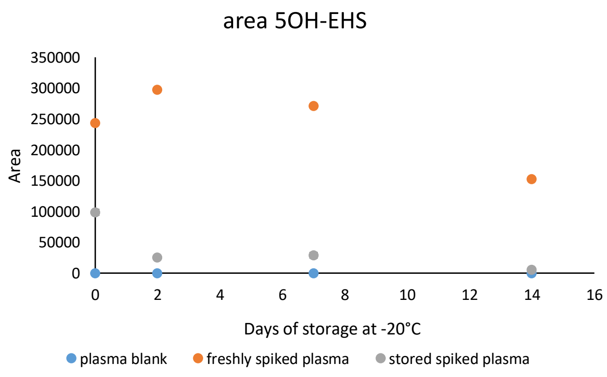

Figure S 4: area of 5OH-EHS in plasma over the course of 14 days.

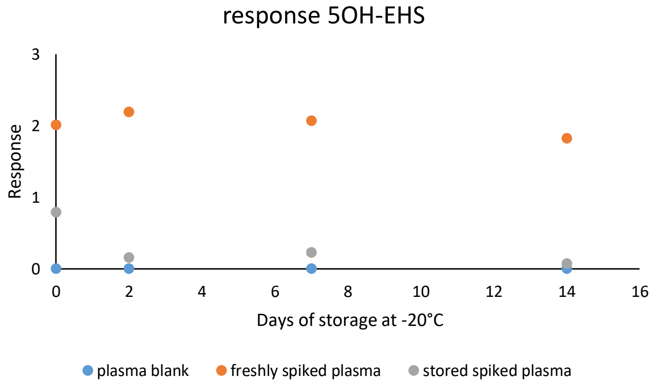

Figure S 5: response of 5OH-EHS in plasma over the course of 14 days.

S6.8. Determination of EHS and its metabolites in human plasma samples

Referring to the work of Klotz et al., it has not yet been possible to establish a sample-preparation procedure for EHS and 5OH-EHS in plasma with a hydrolysis step before protein participation (Klotz et al. 2019). We therefore decided on a different operating order with the protein precipitation first, followed by conjugate hydrolysis after evaporation of the organic solvent, which has been similarly applied by Freiser et al. (Freiser and Jiang 2009). The optimal precipitation was achieved using 600 µL acetonitrile on 300 µL plasma. Acetonitrile was the precipitation solvent of choice for both Klotz et al. and Jiang et al. as well (Jiang et al. 1996; Klotz et al. 2019). The equal extraction of EHS and the EHS glucuronide was tested with this procedure, resulting in absolute recoveries with no significant difference. After the precipitation, the supernatant was evaporated and buffer as well as enzymes were added for hydrolysis, after which the samples were ready for analysis.

Notable findings during the optimization were the importance of the use of glassware vials throughout sample preparation. Low recoveries were observed when using plastic tubes, which could be due to absorption effects.

Following the optimization of the sample preparation, the method was validated. To determine the inter-day precision, pooled-plasma samples were spiked with the analytes and measured over the course of several days. These measurements revealed that the recoveries of 5OH-EHS, 4OH-EHS, 2OH-EHS, 6OH-EHS, 4oxo-EHS, and 5oxo-EHS declined over time. At first, we assumed that analyte adsorption to the plasma proteins caused this effect. Sample preparation was therefore revisited to

optimize protein precipitation towards better analyte desorption from the plasma proteins. Several solvents and combinations (acetonitrile, methanol, both in combination, addition of acid or base) as well as techniques (precipitation speed and vortex time after precipitation) were tested but resulted in no notable improvement in the recoveries. The next consideration was that esterase enzymes in human plasma are still active, even after or during storage at –20 °C (Williams 1987). The cleavage product would be salicylic acid; as a result, stored spiked plasma samples, stored blank-plasma samples, fresh blank plasma samples, and freshly spiked plasma samples were analysed for salicylic acid. All plasma samples were sourced from the same batch of pooled plasma. The measurements revealed elevated salicylic acid areas (by a factor of 4.6) in the spiked and stored samples in comparison to the freshly spiked samples or the stored blank samples over the course of 14 days. Simultaneously, the areas and responses of analytes declined over time. Both observations could be made within the storage period but were most apparent directly after spiking. This outcome supports the hypothesis that ester cleavage takes place. Since the enzyme addition and hydrolysis step was omitted in this experiment because plasma was spiked with unconjugated analytes, an ester cleavage through deconjugation enzymes can be ruled out. However, to determine the exact cause of the ester cleavage, further investigations are needed. An analysis of the plasma samples acquired in the exposure experiment would not yield reliable data and was therefore cancelled. This result is also important for possible future evaluations of EHS and its metabolites in human plasma.

Reference list of the supplementary material:

- Freiser H, Jiang Q (2009) Optimization of the enzymatic hydrolysis and analysis of plasma conjugated gamma-CEHC and sulfated long-chain carboxychromanols, metabolites of vitamin E. Anal Biochem 388(2):260–265. doi: 10.1016/j.ab.2009.02.027
- Jiang R, Hayden CG, Prankerd RJ, Roberts MS, Benson HA (1996) High-performance liquid chromatographic assay for common sunscreening agents in cosmetic products, bovine serum albumin solution and human plasma. J Chromatogr B: Biomed Sci Appl 682(1):137–145. doi: 10.1016/0378-4347(96)00063-1
- Klotz K, Hof K, Hiller J, Göen T, Drexler H (2019) Quantification of prominent organic UV filters and their metabolites in human urine and plasma samples. J Chromatogr B Analyt Technol Biomed Life Sci 1125:121706. doi: 10.1016/j.jchromb.2019.06.033
- Kuhlmann L, Hiller J, Göen T (2024) Comprehensive assessment of the UV-filter 2-ethylhexyl salicylate and its Phase I/II metabolites in urine by extended enzymatic hydrolysis and on-line SPE LC-MS/MS. Talanta 276:126223. doi: 10.1016/j.talanta.2024.126223
- Williams FM (1987) Serum enzymes of drug metabolism. Pharmacol Ther 34(1):99–109. doi: 10.1016/0163-7258(87)90094-5
